# Family TXT: Feasibility and Acceptability of a mHealth Obesity Prevention Program for Parents of Pre-Adolescent African American Girls

**DOI:** 10.3390/children5060081

**Published:** 2018-06-19

**Authors:** Chishinga Callender, Deborah Thompson

**Affiliations:** USDA/ARS Children’s Nutrition Research Center, Department of Pediatrics, Baylor College of Medicine, 1100 Bates Street Houston, TX 77030, USA; Chishinga.Callender@bcm.edu

**Keywords:** obesity, prevention behaviors, home environment, parents, girls, African American, text messages, self-determination theory, feasibility, mHealth

## Abstract

Obesity prevalence is greater in African American girls than their non-Hispanic white peers. Obesity prevention programs are needed to help parents create an obesity-preventive home environment. This paper reports the feasibility and acceptability of a mHealth child obesity prevention program consisting of self-determination theory-grounded text messages promoting a healthy home food and activity environment to parents of 8–10-year-old African American girls. A one-group design with baseline and immediate post-intervention assessments was utilized. Mothers (*n* = 19) received 36 text messages over 12 weeks. Feasibility and acceptability were assessed through staff logs and post-intervention surveys and an interview. Feasibility and acceptability criteria were met. Mothers reported positive reactions to the intervention; they liked the program, used the information, and all but one gave it an A or B grade. The majority made changes and shared the text messages with others. This research provides evidence that a theoretically grounded mHealth child obesity prevention intervention is feasible and acceptable to parents of African American girls.

## 1. Introduction

The obesity epidemic affects African American girls at a disproportionate rate. Between the ages of 6 to 11 years old, prevalence of obesity was 22% in African American girls compared to 14% in non-Hispanic white girls [1]. This pattern continued into adolescence, with 12–19-year-old African American girls having an obesity prevalence of 24% compared to 20% in non-Hispanic white girls [1]. Obesity in childhood often tracks into adulthood [2] and is associated with negative health outcomes, including cardiovascular disease and type 2 diabetes [3]. Thus, finding effective ways to prevent obesity during childhood is an important public health issue.

Several lifestyle behaviors, including diet and physical activity (PA), influence obesity risk [4]. For example, not meeting fruit and vegetable recommendations, consuming a high fat diet, and inadequate PA have been associated with increased obesity risk [5,6,7]. Since childhood dietary and PA choices are likely to continue in adulthood [2], developing healthy diet and PA behaviors in children may be an effective way to prevent adult obesity. Because sedentary behavior, stress and sleep patterns may also contribute to obesity risk [8,9,10], obesity prevention interventions for African American girls should address these behaviors in addition to diet and PA.

Interventions promoting behavior change should be informed by psychological theories [11]. The self-determination theory (SDT) is a theory of motivation and suggests that three basic psychological needs guide motivation and thus influence a specific behavior. These needs include: (1) autonomy (having choice and control); (2) competence (having the knowledge, skills and ability to accomplish a behavior); and (3) relatedness (connection to self and important others) [12]. When these needs are satisfied, there is an integration of the behavior into one’s self-identity which leads to an increase in autonomous (i.e., self-directed) motivation [12].

Parents serve a primary role in creating the home environment [13] and are critical stakeholders in developing and encouraging healthy lifestyle behaviors in their children. The African American Collaborative Obesity Research Network (AACORN) recommends research focusing on strategies to influence parents to reduce childhood obesity risk factors [14]. Therefore, obesity prevention interventions for African American girls should include parents. Previous studies have developed obesity prevention programs for African American girls promoting healthy eating and PA behaviors [15,16,17,18,19,20,21,22,23,24,25]. However, there are few child obesity prevention programs developed specifically for parents of African American girls [26,27,28,29,30]. Because it is important to incorporate culturally appropriate strategies into behavior change interventions to support successful outcomes [31], research is needed to identify ways to address this.

Interventions that incorporate technology, including mobile health (mHealth), have the potential to encourage obesity-preventive behaviors. The Internet and cell phones are used extensively in the African American population. The Pew Research Center reported that 80% of African Americans use the Internet, and 92% own a cell phone [32]. Furthermore, 69% of African American adults, who are Internet users, seek health information online [33]. Research has found that 53% of African American parents utilized the Internet to seek health information [34]. In addition, text messaging is a common communication method used by African Americans. According to the Pew Research Center, 79% of African Americans send and receive text messages [35]. African American parents and women have shown interest in receiving text messages and support the use of text message-based interventions in providing health information [34,36,37,38]. However, we are aware of no studies that have utilized text messages as a primary method for helping parents of pre-adolescent African American girls create a home environment that promotes and supports child obesity prevention.

In addition, previous studies have not examined the feasibility of text messages promoting obesity-preventive behaviors in a parent-focused intervention for this population [39,40,41,42]. An assessment of feasibility is an essential step when creating an untested intervention [43]. Feasibility studies are small-scale tests that assess key aspects of an intervention (e.g., recruitment, attrition, acceptability) prior to the deployment of a fully powered study to assess efficacy [43,44,45]. The purpose of this paper is to report the feasibility and acceptability of a child obesity prevention mHealth program that sent SDT-grounded text messages promoting a healthy home food and activity environment to parents of 8–10-year-old African American girls.

## 2. Materials and Methods

### 2.1. Design

A one-group design was utilized. Data collection occurred at baseline and immediate post-intervention (post 1). The institutional review board at Baylor College of Medicine (H-27505) approved the study protocol.

### 2.2. Study Participants

Inclusionary criteria included being the parent of an 8–10-year-old African American girl, having access to a mobile phone that sends and receives text messages, having Internet access and a personal email address, and a willingness to receive text messages from the study and to participate in data collection. Exclusionary criteria included not having access to a mobile phone that sends and receives text messages and an unwillingness to receive text messages from the study. Parents provided written informed consent prior to participation in the study.

### 2.3. Sample Size

The recruitment goal for the study was 20 parents. Due to a lack of agreement on sample sizes for feasibility studies [46], this sample size was considered sufficient to evaluate feasibility and acceptability.

### 2.4. Recruitment

Parents were recruited from standard methods including the United States Department of Agriculture/Agricultural Research Center (USDA/ARS) Children’s Nutrition Research Center (CNRC) volunteer database and recruitment announcements on websites (i.e., Texas Children’s Hospital). The study coordinator informed parents about the study, described it in detail, and if the parent was interested, screened for eligibility.

### 2.5. Intervention

The 12-week intervention was guided by SDT. Parents received 36 text messages to help them create a home environment that promotes and supports child obesity prevention. Text messages emphasized healthy eating, PA, reduced stress, limited sedentary activity, and adequate sleep. Most of the text messages included links to reputable websites (ChooseMyPlate.gov, EatRight.org, Health.gov). The links were presented in a shortened URL format at the end of the text message. The purpose of the links was to provide interested parents with more information on the topic addressed in the text message (e.g., recipes, free things to do in Houston, kid-friendly yoga routine, tips to reduce screen time, tips to reduce stress). Examples of text messages with links were:

(1) “Check out these recipes for quick and easy family meals.” http://goo.gl/1awttE
(2) “Help your child handle stress with these tips.” http://goo.gl/WTEiqk

For a more detailed description of intervention development and content, please refer to the paper reporting this work [47].

Based on feedback received from parents during development, three text messages per week (2 weekdays, 1 weekend day) were sent. Texts on healthy eating and PA behaviors were sent Tuesday–Friday, and texts on stress, sedentary activity, and sleep were sent on Saturday. Every Monday, parents received an email summary with the three text messages sent the previous week. Records were maintained of texts sent and any technical issues that occurred. The intervention began in July 2017 and ended in October 2017.

### 2.6. Feasibility and Acceptability Criteria 

To make a determination of feasibility and acceptability of this approach, the following criteria were established: recruitment goals (N = 20) met in four months, attrition rate less than 10%, and program (content and approach) deemed acceptable by parents.

### 2.7. Data Collection

Parents participated in baseline and post-intervention data collection. Parents completed self-report questionnaires online. The surveys were hosted on a secure, password-protected website. Parents were emailed a link and a private password to complete the surveys. Surveys assessed standard demographic characteristics (baseline only), parent diet and PA behaviors, psychosocial characteristics, and parent report of child behaviors. In addition, parents completed a 10-item assessment of program components. Sample items included, “In general, what did you think about the text messages”, “Did you use the information in the text messages”, and “Would you recommend this program to other parents”.

After completing the online surveys, a trained interviewer conducted a semi-structured telephone interview with each parent to develop a more in-depth understanding regarding their thoughts about the program and the text messages and suggestions for needed modifications. The interviews were scripted and contained open-ended, non-leading questions; probes and prompts were used to clarify and explore responses. Examples of interview questions included, “Please tell me what you thought about the messages,” “In your opinion, how helpful were the text messages in helping your family make healthy choices, and “What were your favorite text messages.” Each interview was digitally recorded and designed to take no more than one hour to complete. After completion of baseline and post 1 data collection, parents received a $40 money order and a $50 money order respectively. After completion of baseline data collection, parents also received a Fitbit Alta to encourage physical activity.

### 2.8. Feasibility and Acceptability Assessment

Recruitment was monitored through logs maintained by the study coordinator. Attrition rate was determined by the number of participants completing both baseline and post-intervention data collection. Program acceptability was assessed by a 10-item questionnaire assessing program components and through qualitative interviews.

### 2.9. Data Analysis 

Descriptive statistics (frequencies, percentages) were calculated for the demographic characteristics and program acceptability questionnaires. Attrition rate was calculated by dividing the total number of parents who completed post 1 data collection by the total number of families enrolled. Acceptability was assessed by percentages for responses to items on the program acceptability questionnaire and by analyzing qualitative data regarding parent reactions to the program. Consistent with the approach used by others [48], the interviewer created a summary of key points that emerged from the interviews. The summary was utilized to provide insight into parents’ responses to the program acceptability questionnaire, as well as their reactions to the program and thoughts regarding needed changes. To support qualitative findings, verbatim quotes from the interview were included.

## 3. Results

### 3.1. Participant Characteristics 

The majority of mothers were African American (94.7%), 34–43 years old (57.9%), and married (73.7%). Highest level of household education was fairly equally distributed with 31.6% having some college education, 26.3% having a college degree, and 36.8% having post-graduate education. The majority of mothers (52.6%) had a household income greater than $61,000. In addition, the majority of mothers did the food shopping (84.2%) and food preparation (84.2%) in the home (Table 1). All mothers met the inclusionary criteria for having an 8–10-year-old African American daughter.

### 3.2. Feasibility 

All feasibility criteria were met. Twenty mothers were recruited and enrolled in the study. The recruitment sources for the study included the USDA/ARS Children’s Nutrition Research Center (CNRC) volunteer database (N = 16), a recruitment announcement on the Texas Children’s Hospital employee website (N = 2), and parent referrals (N = 2). The recruitment goal was reached in approximately three months (March 2017–June 2017); therefore, recruitment procedures and methods appeared effective for reaching the target audience within a reasonable time period. Of the 20 mothers enrolled in the study, one did not complete baseline data collection and was dropped from the study. The remaining 19 mothers completed baseline and post-intervention data collection, indicating 5% attrition. Thus, the attrition criterion (attrition <10%) was met.

### 3.3. Acceptability

The approach used in this study (mHealth) was found to be acceptable to mothers who participated in the study. All mothers reported liking the text messages; a majority liked the texts a lot (63.2%), and a few liked the texts a little (36.8%). The majority of mothers read a lot of the text messages (84.2%) and shared them with family or friends (63.2%). When asked about the links in the text messages, a majority of mothers reported liking the links a lot (68.4%), and with only a few reporting they liked the links a little (31.6%). More than half of the mothers clicked on a lot of the links (52.6%) and shared the links with family or friends (52.6%). When asked to give the intervention program a grade, the majority of mothers gave it an A (63.2%), followed by a B (31.6%) and a C (5.3%). In addition, all mothers reported using the information in the text messages and recommending the program to other parents (Table 2). Several mothers provided comments about the program in the questionnaire, and those comments are presented in Table 3.

The qualitative data supported the results from the survey assessing reactions to the program. All of the mothers provided positive feedback on the text messages. Overall, they thought the text messages were informative, helpful, good reminders, and easy to access at any time:

*“I found the text messages to be informative, and I liked the variety of information. The other thing that I enjoyed was that it wasn’t all geared towards help your kid not be fat; it was help your kid be healthy.”*

*“I thought they were helpful and it made me conscious to when you would send them. If I didn’t read it right then, when I settled down at night, I’d read it and it helped me to remind my daughter too, like we’d go for a walk or if I didn’t feel like, let’s go for a bike ride, just kept conscious to keep active and eat better.”*

The majority of mothers (*n* = 17) favored the frequency of the text messages. One mother shared, *“I mean I really didn’t complain about the frequency of them. It wasn’t a nuisance. Three is fine. It’s not too many, and it’s not too little.”* Only two mothers recommended changing the frequency to two text messages per week.

Most mothers (*n* = 15) also favored the time of day they received the text messages. One mother shared, *“It was fine because I mean it wasn’t (ever) too early, and it wasn’t too late in the day.”* Only four mothers suggested changing the time of day the messages were received to the morning, lunchtime, or between late morning and early afternoon. All of the mothers favored the links in the text messages. Words used to describe the links included “informative,” “helpful,” “good,” “fine,” and “easy access.” Mothers shared:

*“It was just right. It was always one single text, never more than one and the shortened URL was really good. I liked that I could copy and share it easy.”*

*“I loved the links. I think the links were definitely the most helpful…Yeah, it was one thing to have the text message, but I utilized the links more than anything else because usually it was information I didn’t have or it was a reinforcement of information.”*

Only one mother experienced an issue with accessing the links on her phone at the beginning of the intervention. The reason for the issue is undetermined as the participant suggested it may or may not have been her mobile phone carrier.

When mothers were asked about their favorite text messages, they reported the following categories that included their favorite texts: healthy eating, PA, stress and sleep. Healthy eating messages liked by the mothers included recipes, meal planning, and substitutions:

*“I like the one about changing a sugary snack to something healthy. I like the replacement part of it.”*

*“The other one was definitely the meals or like the helpful hints of meal prep…that kind of thing…cause that’s easier for me to have at the fingertip…look it’s in a text, I don’t have to download an app…Those were helpful.”*

PA messages liked by the mothers included reminders to get up and be active and activities to do with kids inside and outside of the home. More specifically, mothers enjoyed receiving the text messages on the local parks and fun, free activities to do in the Houston area:

*“The ones about, like I said, when I received the messages about the stuff to do around Houston, like the fun free things you can do, all the parks and nature things like to get out and exercise. Because anything that allowed me and my girls to be active, cause they like to be active and they [are] doing things, that’s always a plus for me.”*

Reasons for selecting the text messages included looking for new ideas, liking the suggestions offered, reminders to practice behaviors, working on practicing the behavior, needing help in a particular area, increased awareness to practice a behavior, motivation for the family, and an opportunity for the mother and daughter to participate in an activity together.

When mothers were asked about their least favorite text messages, the majority of mothers (*n* = 15) reported not having a least favorite text message. Mothers described the content of the messages as being “informative,” “helpful,” “educational” and “appropriate.” One mother shared, *“I can’t really say. Yeah, I mean I didn’t really have any that was oh, I don’t like that or it wasn’t appropriate. I was okay with all of them.”* Of the four mothers who had a least favorite text message, their reasons for this included not making any changes for the given category (sleep, stress), hearing similar information from the pediatrician, children already participating in activities, and not wanting to be reminded of a behavior they should be practicing (sleep).

When mothers were asked, “how helpful were the text messages in helping you help your family make healthy choices,” all the mothers expressed that the text messages were helpful. They liked the information provided in the messages. The information in the messages served as reminders of healthy behaviors. The messages helped the mothers to be conscious of practicing healthy behaviors and helped them to stay on track in practicing healthy behaviors:

*“It was very good, and in terms of the information that was being given, it helped me to stay focused on those preparation meals, instead of…fast food.”*

*“I think they were more reminders of good, healthy behavior, and so it brought it to the forefront of my mind.”*

Furthermore, the mothers liked the ease and convenience of the text messages. More specifically, mothers liked having easy access to the messages and being able to read the text messages at their own pace. When asked what they liked about the text messages, mothers shared:

*“If I didn’t read it right away, I got another chance at it...knowing that I was given a chance to read something without being bombarded at one time.”*

*“That I could always go back to them, and they didn’t disappear unlike something in my email. It was real easy and convenient ‘cause I just go to my messages and click on the link.”*

When asked about any changes made because of the text messages, most mothers (*n* = 15) made changes and reiterated making a conscious choice to practice healthy lifestyle behaviors. Changes were based on healthy eating, PA, less screen time, and sleep:

*“Changed some of our eating habits, a little less sugar, to more fruits and vegetables…we did the less TV time and picked up a jump rope, and she’s playing soccer now.”*

*“…it more helped me with the physical activity like the websites and then also coming down to when I get home to put on a YouTube exercise video or take extra steps and even the reminder to utilize the outside parks in the middle of the week if you have time.”*

*“Well, I started reducing my daughter’s TV time, and she started helping me like make vegetables and stuff like that… Yeah, the sleep patterns improved a lot. Yeah, she feels better…she wakes up better in the mornings.”*

*“I made a conscious effort to make sure that I had fruit out on the table every day when my daughter got home. More exercise, like we went walking, walked more and I let her ride her bike more often.”*

Of the four mothers who made no changes as result of the text messages, some shared that the text messages improved the healthy lifestyle behaviors they were already putting into practice. One mother shared, *“I can’t think of anything like that we really changed. Like I said, because we were kind of really already focusing on exercising and trying to eat properly and all of those types of things. We were kind of already were doing those things. I don’t think it really changed anything more than just maybe enhanced.”*

When asked, “should any of the text messages be eliminated from future studies,” all of the mothers emphasized that none of the text messages should be eliminated from future studies. Mothers commented that the messages were “helpful,” “useful,” “relevant,” “informative” and “appropriate.” When asked, “should any of the text messages be added to future studies,” mothers recommended adding text messages with more recipes, including vegan and vegetarian meals and kid-friendly infographics demonstrating how to make a dish. They also recommended including text messages on drinking more water and the consequences of poor healthy eating choices like diabetes. Mothers also suggested incorporating texting messages on all of the behaviors, and some mothers emphasized that text messages on healthy eating, physical activity and stress should be included in future studies. One mother shared, *“The information about stress…because stress affects all aspects, no matter what; it’s not like…it’s not just isolated, stress affects every condition, so that’s good info to always have out there, put out there.”*

The interviews revealed that the mothers read most or all of the text messages. The interviews also revealed that a majority of the mothers shared the text messages with family or friends. Persons they shared the information with included sisters, friends, their own mothers, their children, neighbor with children, sister-in-law, cousin, husband and Girl Scout troop. One mother also shared the text messages with people via social media. When asked, “who could benefit from these text messages,” more than half of the mothers shared that everyone could benefit from the text messages. Some mothers shared that the text messages would be helpful and applicable to everyone regardless of age. For example, one mother stated, *“I think everyone can benefit as far as…I mean the text messages [are] basically for kids and adults. I think anyone…all the text messages were very beneficial and positive in some kind of way or form.”*

The majority of mothers (*n* = 16) used text most often to read the messages. Reasons included that texts were “easy,” “accessible,” “fast” and “instant.” One mother shared, *“Text because it was easy for me to find like I received it and I checked the message at the time, and then clicked on the links if I needed to. It was just easier. Whereas with my email, I have so many different emails. I mean I have two on my phone, but yeah, sometimes I miss them, and that’s why I prefer text.”*

A few mothers (*n* = 3) used both text and email to read the text messages. Reasons included being able to share the text messages with others, being reminded about the text messages received, and easier to read. For example, one mother shared, *“I used both, but I used email. Because like I said, the text was easy access, but with email you can see but with text you would be depending on your phone provider. In the email you can see better, you can click on the PDF and it comes right up.”*

## 4. Discussion

This study demonstrated the feasibility and acceptability of a mHealth intervention to help parents of pre-adolescent African American girls create a healthy home environment that promotes and supports child obesity prevention. Findings revealed that mothers liked receiving text messages promoting a healthy home food and activity environment and found them helpful. Overall, acceptability of the structure and content of the text messages was high. This is similar to findings by previous studies. The comments on the text messages as informative, helpful and good reminders confirm feedback provided by mothers in the formative phase of the study [47]. A study by Downing et al. found that parents were positive about the text messages [40]. More specifically, parents reported text messages were an easy and convenient method to receive the information, and the frequency and the content of the links were acceptable by the parents [40]. These findings were similar to our sample as the mothers reported liking the ease and convenience of the texts, the majority accepted the frequency of the texts, and all accepted the content of the links in the texts.

Furthermore, the acceptance of the link content and format by mothers in the pilot study confirms the feedback of mothers in the formative study [47]. Mothers reported they liked the links and would click on the links in the formative phase, and our current sample reported liking and clicking on the links. All mothers in our recent study would recommend the program to other parents. This is similar to findings by Militello et al. [41], which found that 100% of parents in a parent-focused text messaging-based intervention for overweight/obese preschoolers would recommend the program to other parents of preschoolers. Hashemian et al. [42] also reported that 96.7% of mothers in a text messaging program to improve oral health knowledge would recommend the program to family and friends.

The mothers in our study recognized that text messages were useful and helpful. All mothers reported that they used the information in the text messages. This is similar to findings in a recent study in which parents reported that the text messages were extremely or very useful [40]. In addition, a majority of mothers in the text messaging intervention group of the oral health study reported that the text messages were useful [42]. In our qualitative interviews, all mothers noted that the text messages were helpful in helping them help their family make healthy choices. The majority of African American women, in a mHealth intervention promoting PA, reported that the text messages were helpful to very helpful in promoting PA [37].

Mothers reported they made changes as a result of the text messages. This is similar to findings by Downing et al., which found that the program helped parents change their approach and being more conscious of sedentary behavior and screen time [40]. Mothers in our study reported changing behaviors related to healthy eating, PA, sedentary behavior, sleep and stress and acknowledged being more conscious or aware of practicing obesity-preventive behaviors in the home environment. A previously mentioned study reported that a majority of the participants read at least 9 of the 12 behavioral text messages [40]. This outcome supports both the program acceptability assessment and qualitative interview responses as most mothers in our study reported reading a lot of the messages.

The pilot study was conducted to examine the feasibility and acceptability of the text message-based intervention designed to help parents of 8–10-year-old African American girls develop a healthy home food and activity environment that promotes and supports child obesity prevention. Results indicate both the feasibility and acceptability of this approach.

The study has several limitations. The majority of mothers were married and of higher income and education. Twelve weeks may not have been long enough to achieve sustained change in targeted behaviors. Mothers’ self-report of behaviors may under- or over-report actual behavior. The sample size was small and participants were from only one geographic region (Southwestern) in the United States, which limit generalizability of the findings. However, the purpose of this study was to evaluate the feasibility and acceptability of this approach, which typically do not utilize large, diverse samples.

Future research is needed to assess intervention effects on parent and child behaviors. Future research should also explore the effect of text messages tailored to the needs and preferences of each parent (i.e., frequency, time of day, content received). Finally, research should test the additive effects of sending text messages to other family members (e.g., grandparents, other close relatives) on the home food and activity environment and child diet and PA behaviors. These studies could expand the literature on mHealth child obesity prevention interventions designed for African American families.

## Figures and Tables

**Table 1:** Descriptive statistics of parent characteristics for the Family TXT Study (*n* = 19).

Parent Characteristics	*n*	Percentage
Gender		
Male	0	0.00
Female	19	100.00
Race/Ethnicity		
African American	18	94.74
Hispanic/White	1	5.26
Age (Years)		
30–39	7	36.84
40–49	11	57.89
50–59	1	5.26
Marital Status		
Married	14	73.68
Non-married	5	26.32
Highest household education		
Technical school	1	5.26
Some college	6	31.58
College graduate	5	26.32
Post graduate study	7	36.84
Household income		
<$21,000	1	5.26
$21,000–$41,000	3	15.79
$41,000–$61,000	5	26.32
>$61,000	10	52.63
Children under age of 18 living in home		
1	3	15.79
2	7	36.84
3	6	31.58
4	2	10.53
5	1	5.26
Adults living in home (not including self)		
0	3	15.79
1	10	52.63
2	5	26.32
3	1	5.26
Food shopping in the home		
Me	16	84.21
Another adult	1	5.26
Shared responsibility	2	10.53
Food preparation in the home		
Me	16	84.21
Another adult	0	0.00
Shared responsibility	3	15.79

**Table 2:** Reactions to text messages (*n* = 19).

Parent Responses	*n*	Percentage
What did you think about the text messages?		
Liked a lot	12	63.16
Liked a little	7	36.84
How many did you read?		
A lot	16	84.21
Some	2	10.53
A few	1	5.26
Did you share the text messages with family or friends?		
Yes	12	63.16
No	7	36.84
Did you use the information in the text messages?		
Yes	19	100.00
No	0	0.00
What did you think about the links in the text messages?		
Liked a lot	13	68.42
Liked a little	6	31.58
How many links did you click on?		
A lot	10	52.63
Some	4	21.05
A few	5	26.32
Did you share the links with family or friends?		
Yes	9	47.37
No	10	52.63
What grade would you give Family TXT?		
A	12	63.16
B	6	31.58
C	1	5.26
Would you recommend this program to other parents?		
Yes	19	100.00
No	0	0.00

**Table 3:** Parent post-intervention survey comments.

Very informative about changing your life style and helping your child be healthy
I enjoyed the information I received and the delivery method made it easy to share with others. I liked having the option of either forwarding a text or sharing the link on Facebook.
Overall really good information on healthy food and activities for in-doors and outside. Lots of family activities.
Wonderful program! The links were great reminders and some taught me new things. I would think on the information and process how to incorporate the suggestions in our life! Thank you for allowing me to be a part of this study!
As a busy mother of three, sometimes I get caught up in daily life and activities, I tend to forget important things like nutrition and healthy eating habits. I feel like this has helped me be accountable and reminded me to do more activities with my family.
I liked receiving the information about health risk, diets, exercising, and healthy food ideas. I knew most of this information already I just reiterated what I already knew and used this information for my family’s best interest.
This has been an eye opener. I have read and learn about exercise and food-healthy food and food habits!
I enjoyed the program. It gave me reminders to keep with my healthy eating and exercise initiative! I need a little push here and there and I definitely needed the reminders! I would have liked a little more interactive messages.
